# An Increase in Mean Aortic Valve Gradients the Day After Transcatheter Aortic Valve Implantation: The Effects of Evolving Anesthesia Techniques

**DOI:** 10.3390/jcm14103272

**Published:** 2025-05-08

**Authors:** Benjamin Fogelson, Raj Baljepally, Billy Morvant, Terrance C. Nowell, Robert Eric Heidel, Steve Ferlita, Stefan Weston, Aladen Amro, Zachary Spires, Kirsten Ferraro, Parth Patel

**Affiliations:** 1 Division of Cardiology, Department of Medicine, University of Tennessee Graduate School of Medicine, Knoxville, TN 37920, USA; rbaljepa@utmck.edu; 2 Department of Anesthesia, University of Tennessee Graduate School of Medicine, Knoxville, TN 37920, USA; smorvant1@utmck.edu (B.M.); tcnowell1@utmck.edu (T.C.N.); 3 Department of Surgery, University of Tennessee Graduate School of Medicine, Knoxville, TN 37920, USA; rheidel@utmck.edu; 4 Division of Internal Medicine, Department of Medicine, University of Tennessee Graduate School of Medicine, Knoxville, TN 37920, USA; sferlita@utmck.edu (S.F.); sweston@utmck.edu (S.W.); aamro@utmck.edu (A.A.); zspires1@utmck.edu (Z.S.); kferraro@utmck.edu (K.F.); ppatel6@utmck.edu (P.P.)

**Keywords:** transcatheter aortic valve implantation, transvalvular gradient, anesthesia, general anesthesia, monitored anesthesia care, dexmedetomidine, propofol

## Abstract

**Background and Objectives**: After transcatheter aortic valve implantation (TAVI), transvalvular gradients increase immediately following the procedure up to 24 h afterward. While factors such as anesthesia type and fluid status have been suggested as potential contributors, the underlying cause remains unclear. With advancements in TAVI techniques, there has been a shift in anesthesia protocols from general anesthesia (GA) to monitored anesthesia care (MAC). This study aimed to assess the impact of GA and MAC on the increase in transvalvular gradients observed 24 h post-TAVI. **Methods**: A retrospective, single-center analysis was conducted on patients who underwent TAVI at our institution between 2011 and 2023 (n = 744, males = 421). The patients were divided into two groups: those who received GA (n = 201) and those who received MAC (n = 543). The GA group received either inhaled anesthetics, with or without propofol infusions, or propofol infusions at a rate of ≥100 mcg/kg/min. The MAC group received bolus doses and continuous infusions of dexmedetomidine. Transvalvular gradients were compared between immediate and 24 h post-procedure echocardiograms. **Results**: The average age of patients in the GA group (78 years [IQR 71–83]) was similar to that of the MAC group (77 years [IQR 71–83]). The GA group had a higher prevalence of comorbidities at baseline. Both groups exhibited stable, normotensive blood pressure levels during the procedure, though the GA group required more vasopressors and intravenous fluid. The GA group showed a 24 h post-TAVI mean transvalvular gradient change of +5.1 mmHg [IQR 3–8.1], while the MAC group had a 24 h mean transvalvular gradient change of +5.8 mmHg [IQR 3.2–9], with no significant difference between the groups (*p* = 0.139). **Conclusions**: Despite the greater cardiovascular depressive effects and increased need for vasopressors and fluid resuscitation in the GA group, there was no significant difference in the increase in transvalvular gradients between the GA and MAC groups at 24 h post-TAVI. Further research is needed to fully understand the reasons behind the increase in gradients observed after TAVI.

## 1. Introduction

Transthoracic echocardiogram (TTE) plays a crucial role throughout the transcatheter aortic valve implantation (TAVI) process, from procedural planning to long-term follow-up. Immediately after TAVI, TTE is used to assess valve placement and function and detect complications. Key measurements like mean and maximum transvalvular gradients are critical for evaluating the TAVI function [1,2]. While some studies have linked elevated post-procedural transvalvular gradients to worse outcomes and higher mortality, others have not found a direct correlation [3,4,5]. When comparing immediate post-TAVI to 24 h follow-up TTEs, there is typically a notable increase in transvalvular gradients. Previous studies have suggested that this transvalvular gradient increase can be attributed to a low-flow state induced by anesthetics utilized during the TAVI procedure [6,7].

TAVI has been conventionally performed under general anesthesia (GA) with endotracheal intubation, similar to surgical aortic valve replacement (SAVR). More recently, there has been a notable shift away from GA and towards monitored anesthesia care (MAC) for TAVI [8,9,10,11]. The transition to MAC was prompted by studies demonstrating similar or improved clinical outcomes, including shorter hospital stays, reduced vasoactive medication use, and lower rates of post-procedure delirium compared to GA [10,12,13,14,15,16]. GA was found to have more pronounced hemodynamic effects, including systemic vasodilation and myocardial depression, on the TAVI patient population compared to the anesthetic agents used in MAC [17,18,19].

The most common infused agents used for MAC during TAVI are dexmedetomidine and propofol, respectively. While both agents impact cardiovascular physiology, dexmedetomidine is often favored due to its ability to provide sedation with minimal respiratory depression and a more stable hemodynamic profile compared to propofol, which is associated with greater reductions in systemic vascular resistance and myocardial contractility. While many institutions continue to utilize bolus doses of propofol during the TAVI procedure, dexmedetomidine has become the preferred infused agent for MAC.

Although prior studies have suggested that anesthesia may induce a low-flow state contributing to an increase in transvalvular gradients at 24 h post-TAVI, no independent studies have directly compared the effects of GA versus MAC on these gradient changes to the best of our knowledge. Given the more pronounced cardiovascular depressive effects of GA, we hypothesized that patients receiving GA for TAVI would experience a greater 24 h transvalvular gradient change compared to those receiving MAC.

## 2. Materials and Methods

### 2.1. Study Design

A retrospective analysis was performed on patients who had undergone TAVI at our academic medical institution between January 2011 and December 2023. The study was approved by our institutional review board (IRB Registration number 00005012, approved 1 March 2023). Informed consent was waived by our institution’s IRB, given the retrospective nature of this study. The study was conducted in compliance with the ethical standards of our institution as well as the revised Helsinki Declaration.

### 2.2. Patient Population

Patients with severe aortic stenosis who underwent TAVI between January 2011 and December 2023 at our institution were included in this study. Patients in the study underwent a standardized evaluation by our multidisciplinary Heart Team prior to TAVI. All patients in the study received Edwards SAPIEN balloon-expandable valves. Patients who were excluded from the study included those who underwent concomitant mitral valve intervention or had existing mechanical/bioprosthetic valves. Patients in the study were required to have a TTE immediately after and within 24 h post-TAVI.

### 2.3. Anesthesia and Hemodynamic Details

Procedure reports and anesthesia documentation were reviewed for vital signs, the use of inhaled anesthetics, medication dosages, the use of vasopressors, and peri-procedural intravenous fluid administration.

Patients were divided into two groups for this study: those who received GA and those who received MAC for TAVI. Group allocation was determined by a multidisciplinary team, including cardiologists, anesthesiologists, and a biostatistician, based on the following criteria for GA and MAC. GA was considered when loss of consciousness was induced, during which patients could not be awakened, even by painful stimuli. This was achieved using inhalation anesthetics, with or without the infusion of propofol, or via propofol infusion alone at an initial rate of at least 100 mcg/kg/min. Propofol was titrated based on the bispectral index (BIS), which is a filtered EEG monitor commonly used by anesthetists during total intravenous anesthetics. Patients who were placed in the MAC group were patients who received moderate-to-deep conscious sedation. For the purposes of this study, all patients in the MAC group received an infusion of dexmedetomidine at the following dosages: a loading infusion of 0.5–1 mcg/kg over 10 min for the initiation of procedural sedation followed by 0.2 to 1 mcg/kg/hour for the maintenance of procedural sedation and titrated based on patient tolerance of the procedure and hemodynamic effects. Notably, both groups may have received low-dose boluses of propofol (less than 75 mg in total throughout the procedure); however, these small boluses were considered an amount that would not affect the patient’s overall hemodynamics in the MAC group, given the low total dose and typical half-life of fewer than ten minutes for a bolus dose of propofol. Additionally, patients in both groups may have received intravenous midazolam and intravenous fentanyl pre- or peri-procedurally, which were reported as total doses. Notably, patients who required crossover from the MAC to GA were excluded from the study.

Hemodynamics and hemodynamic support were compared between the two groups. Pre-procedural, immediate post-procedural and 24 h post-procedural blood pressures were compared between the two groups. The use of vasopressors was documented for each group, as well as the total amount of intravenous fluids administered during the procedure. The need for vasopressors and intravenous fluid administration was used as a marker of hemodynamic instability and support during TAVI. Blood pressure, pre- and post-TAVI weights, and net fluid balance were used as a surrogate of volume status.

### 2.4. Echocardiographic Measurements

All echocardiograms were interpreted following the guidelines set by the American Society of Echocardiography (ASE). Pre-TAVI TTE was performed to evaluate the severity of aortic stenosis. Using continuous-wave Doppler, the peak velocity across the aortic valve was measured in both the apical 5-chamber and apical long-axis view. The aortic valve area was calculated using the continuity equation. The left ventricular outflow tract (LVOT) diameter was measured from the inner edge to the inner edge in the parasternal long-axis view with zoom. LVOT velocity was assessed using pulse-wave Doppler in the apical long-axis view. For both immediate and 24 h post-TAVI TTEs, the peak gradient, mean gradient, and continuous-wave velocities were measured in the apical 5-chamber and/or apical long-axis views. For patients who were in atrial fibrillation, their measurements were averaged over three beats.

### 2.5. Endpoint Measurements

The primary outcome assessed in this study was the numerical change (delta) in mean transvalvular gradients immediately and 24 h post-TAVI.

### 2.6. Statistical Analysis

The GA and MAC groups were compared on non-normal continuous and ordinal level outcomes using Mann–Whitney U tests. Medians (Mdn) and interquartile ranges (IQRs) were reported for each group to give context to the group comparisons. Categorical parameters and outcomes were compared between the groups using chi-square tests or Fisher’s exact tests, as needed. Frequency (f) and percentage (%) statistics were presented and interpreted for the comparison of categorical variables. Statistical significance was assumed at a two-sided alpha value of 0.05, and all analyses were performed using SPSS Version 29 (IBM Corp.: Armonk, NY, USA).

## 3. Results

### 3.1. Baseline Clinical Characteristics

The study included 744 patients who underwent TAVI with an Edwards Sapien valve at our academic institution. The patients were divided into two groups, with 201 patients in the GA group and 543 patients receiving MAC. A total of 13 patients were excluded due to the crossover from MAC to GA, including 5 patients due to agitation or discomfort and 8 patients who required vascular surgery at the femoral access site. The median age was similar between the GA and MAC groups (Mdn = 78 [IQR: 71–84] vs. Mdn = 77 [IQR: 71–83] years, *p* = 0.335), and there were no significant differences in gender distribution (58.7% male in GA vs. 55.8% in MAC, *p* = 0.48). The GA group had a higher prevalence of comorbidities, including diabetes mellitus (52.2% vs. 41.3%, *p* = 0.008), hypertension (93.5% vs. 87.5%, *p* = 0.02), peripheral vascular disease (46.3% vs. 18.2%, *p* < 0.001), stroke/TIA (19.4% vs. 11.4%, *p* = 0.004), and chronic kidney disease (60.2% vs. 38.2%, *p* < 0.001). Additionally, the GA group had a higher CHA2DS2-VASc score (Mdn = 5 [IQR: 4–6] vs. Mdn = 4 [IQR: 3–5], *p* < 0.001) with more atrial fibrillation (36.3% vs. 27.7%, *p* = 0.02) and a greater proportion of patients with NYHA class III-IV symptoms (92% vs. 81.1%, *p* = 0.004). The baseline clinical characteristics are summarized in Table 1.

### 3.2. Hemodynamics and Anesthesia

Hemodynamic parameters revealed that pre-TAVI systolic blood pressure was similar between the groups, but diastolic blood pressure was mildly lower in the GA group (Mdn = 65 mmHg [IQR: 57–74] vs. Mdn = 71 mmHg [IQR: 63–82], *p* < 0.001). Immediately post-TAVI, the GA group had higher immediate systolic (Mdn = 128 mmHg [IQR: 114–144] vs. Mdn = 124 mmHg [IQR: 112–139], *p* = 0.017) and lower diastolic blood pressures (Mdn = 53 mmHg [IQR: 46–64] vs. Mdn = 59 mmHg [IQR: 51–67], *p* < 0.001). At 24 h post-TAVI the GA group had lower systolic (Mdn = 126 mmHg [IQR: 117–139] vs. Mdn = 131 mmHg [IQR: 118–142], *p* < 0.001) and diastolic pressures (Mdn = 57 mmHg [IQR: 50–64] vs. Mdn = 63 mmHg [IQR: 56–70], *p* < 0.001).

The GA group received more peri-procedural fluid (Mdn = 700 mL [IQR: 300–950] vs. Mdn = 500 mL [IQR: 250–700], *p* < 0.001) and required vasopressors more frequently (79.1% vs. 35.6%, *p* < 0.001). The administration of medication differed between the groups: the GA group received higher doses of fentanyl (Mdn = 250 mg [IQR: 150–500] vs. Mdn = 0 mg [IQR: 0–75], *p* < 0.001) and midazolam (Mdn = 2 mg [IQR: 0–3] vs. Mdn = 0 mg [IQR: 0–1], *p* < 0.001). Hemodynamics and anesthetic details are presented in Table 2.

### 3.3. Echocardiographic Characteristics

**Figure 1:**
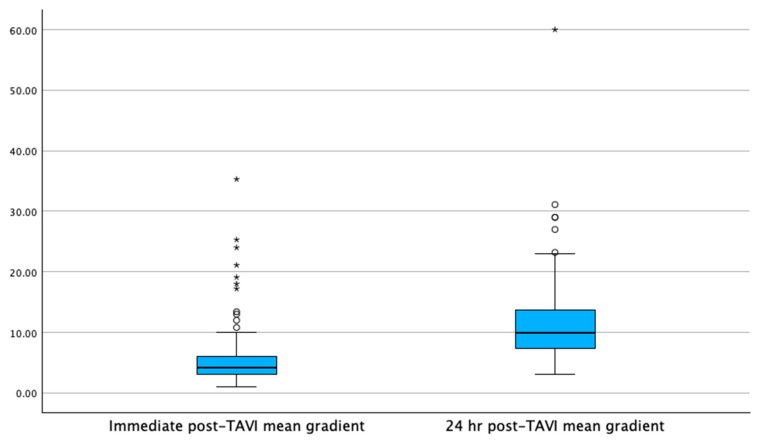
The mean gradient increases at 24 h post-TAVI for the GA group, demonstrating a statistically significant increase (*p* = <0.001). Note: Circles denote outliers at 1.5× the interquartile range; asterisks denote outliers at 3.0× the interquartile range.

**Figure 2:**
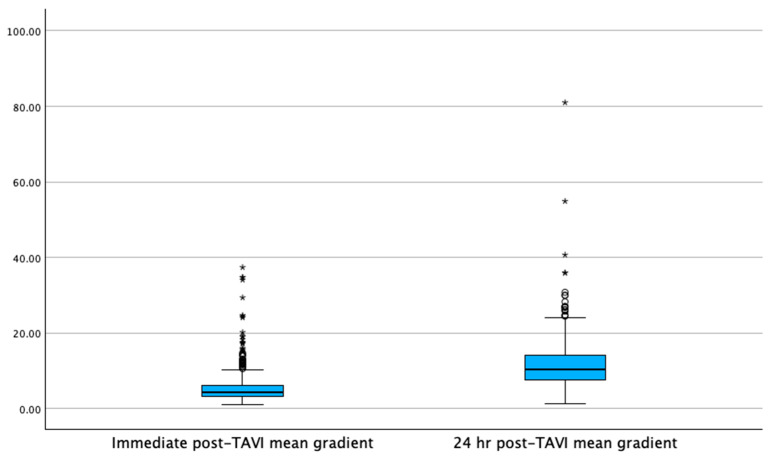
The mean transvalvular gradient increases at 24 h post-TAVI for the MAC group, demonstrating a statistically significant increase (*p* = <0.001). Note: Circles denote outliers at 1.5× the interquartile range; asterisks denote outliers at 3.0× the interquartile range.

**Figure 3:**
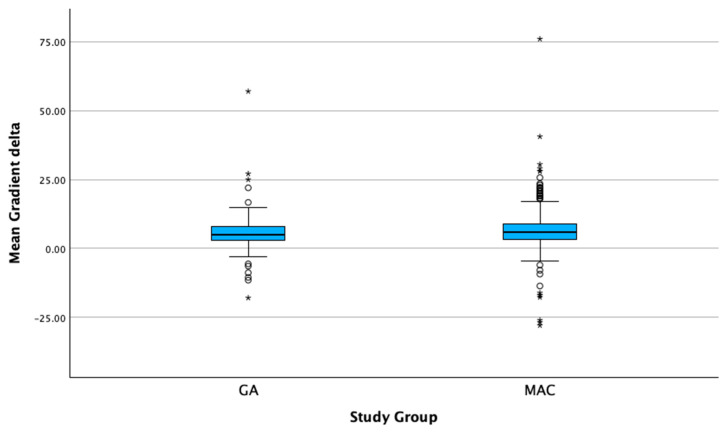
The mean transvalvular gradient change (delta) at 24 h post-TAVI, comparing the GA and MAC groups, demonstrating no statistically significant difference (*p* = 0.139). Note: Circles denote outliers at 1.5× the interquartile range; asterisks denote outliers at 3.0× the interquartile range.

Pre-TAVI left ventricular ejection fraction (LVEF) was lower in the GA group compared to the MAC group (Mdn = 55% [IQR: 50–60] vs. Mdn = 60% [IQR: 55–65], *p* < 0.001). This difference persisted at 24 h post-TAVI, with the GA group showing a lower LVEF (Mdn = 56% [IQR: 53.4–60] vs. Mdn = 61.4% [IQR: 56–66], *p* < 0.001). In contrast, the pre-TAVI aortic valve area and mean gradients were similar between the groups, with no significant differences in immediate post-TAVI transvalvular mean gradients (Mdn = 4.2 mmHg [IQR: 3–6] in GA vs. Mdn = 4.4 mmHg [IQR: 3.2–6.1] in MAC, *p* = 0.21). At 24 h post-TAVI, transvalvular mean gradients remained comparable between the groups (Mdn = 10 mmHg [IQR: 7.4–13.7] in GA vs. Mdn = 10.4 mmHg [IQR: 7–13] in MAC, *p* = 0.192). The increase in means gradients at 24 h was statistically significant in the GA group (+5.8 mmHg, *p* = <0.001) and in the MAC group (+6 mmHg, *p* = <0.001), as shown in Figure 1 and Figure 2. However, the change in delta for the gradients immediately compared to 24 h post-TAVI was not statistically significant between the groups (Mdn = 5.1 mmHg [IQR: 3–8.1] in GA vs. Mdn = 5.8 mmHg [IQR: 3.2–9] in MAC, *p* = 0.139), as shown in Figure 3. Notably, the GA group had a significantly higher prevalence of trace-mild paravalvular leaks post-TAVI (18.9% vs. 5%, *p* < 0.001); however, there was no difference in moderate–severe paravalvular leaks (0.5% vs. 0.2% *p* = 0.47). Echocardiographic data are presented in Table 3.

## 4. Discussion

The main findings of the study were as follows: 1. Patients in the GA group were found to have more baseline comorbidities compared to the MAC group. 2. Both groups had similar, normotensive immediate and 24 h post-procedural blood pressures; however, the GA group required more vasopressor support and fluid resuscitation compared to patients who received MAC. 3. Patients in both groups had a statistically significant increase in transvalvular gradients at 24 h post-TAVI. 4. Although patients who received GA experienced more pronounced cardiovascular depression and required higher amounts of vasopressors and fluid resuscitation, the difference in transvalvular gradient change (delta) both immediately post-TAVI and 24 h post-TAVI was not statistically significant when compared to the MAC group.

While transvalvular gradients have been noted to increase 24 h following TAVI, few studies have investigated the etiology of this phenomenon. It has been hypothesized that various factors inducing a low-flow state may contribute to this observation. For patients undergoing TAVI, the requirement to be nil per os (NPO) prior to the procedure can lead to dehydration and reduced circulating blood volume, potentially contributing to a low-flow state [7]. Additionally, rapid pacing during the procedure has been associated with transient ischemia, which may further exacerbate low-flow conditions [7]. However, large studies specifically quantifying the effects of the NPO status and rapid pacing-induced transient ischemia on increases in the transvalvular gradient increases remain limited. Prior research has also suggested that anesthetic agents and peri-procedural medications with cardio-depressive effects may play a role in transvalvular gradient changes [6,7]. The primary focus of our study was to compare the effects of GA and MAC on 24 h post-procedural transvalvular gradient changes.

Traditionally, TAVI was performed under GA to ensure complete sedation and immobilization during the procedure. Following FDA approval, TAVI was performed for high-risk patients with severe symptomatic AS who were deemed unsuitable for SAVR. Patients at high risk were considered those of advanced age and/or those with multiple comorbidities, and thus, the GA approach ensured an optimal procedural environment. The current study found that patients in the GA group had a greater burden of comorbidities compared to those in the MAC group. This finding aligns with the historical preference for GA in higher-risk patients. In addition, the higher rate of paravalvular leak observed in the GA group is likely related to the earlier era in which these procedures were performed when first-generation TAVR valves were more commonly used. These earlier devices lacked sealing skirts and were associated with greater paravalvular leak risk. Over time, improvements in valve design, operator experience, and the transition from echocardiography to CT-based annular sizing contributed to significantly reduced paravalvular leak rates. As TAVI has evolved and expanded to a broader patient population, anesthetic techniques have also shifted, favoring MAC due to its association with reduced procedural time, faster recovery, and fewer hemodynamic disturbances. These historical trends and evolving clinical practices have led to investigations into the comparative outcomes of GA and MAC in TAVI patients.

The effects of GA and MAC in patients undergoing TAVI have been previously compared [10,11,12,13,14,15,16,17,18,19]. Multiple studies have found that MAC decreases procedure duration, recovery time, and hospital length of stay compared to GA [12,18,20,21,22,23,24]. While some studies have demonstrated that patients receiving MAC for TAVI had lower mortality rates and improved clinical outcomes compared to GA [10,12,13,14,15,16], other studies have found no significant differences in outcomes [25,26,27,28]. Although data regarding outcome superiority remains mixed, it is widely accepted that GA induces more profound cardiovascular depression than MAC. Prior studies have suggested that the hemodynamic effects of GA could lead to a transient low-flow state, potentially contributing to lower immediate post-TAVI transvalvular gradients [6,7]. If this hypothesis were accurate, patients receiving MAC, which preserves hemodynamics more effectively, would be expected to have a lower 24 h increase in transvalvular gradients.

The association between anesthesia type and acute kidney injury (AKI) in TAVI patients further supports the hypothesis that GA induces transient hypoperfusion. Previous studies have shown higher AKI rates in patients receiving GA, likely due to intraoperative hypotension and reduced renal perfusion pressure [18,29]. While we anticipated that the greater cardio-depressive effects of GA would correlate with lower immediate post-TAVI gradients and, consequently, a larger 24 h increase, the similar transvalvular gradient changes between the groups suggested additional contributing factors. Despite statistically significant differences in intraoperative hemodynamic support requirements between the groups, both maintained normotensive blood pressures post-TAVI, with only minor variations in the immediate and post-procedural periods. The increased need for vasopressors and fluid resuscitation in the GA group suggests more frequent episodes of transient hypotension and hypoperfusion. However, this did not translate into differences in transvalvular gradient changes when comparing the GA and MAC groups, raising questions about the primary drivers of this phenomenon.

The authors have three key considerations for the comparable 24 h post-TAVI transvalvular gradient changes observed between the two groups. First, patients in the GA group required significantly more vasopressor support and a greater volume of resuscitation, likely to counteract the vasodilatory and myocardial depressive effects of GA. Despite these intraoperative differences, post-TAVI blood pressures remained within a normotensive range in both groups, with minimal variations. This suggests that prompt and effective hemodynamic management by anesthesia teams can mitigate the potential impact of hypotension and hypoperfusion. Given the transient nature of these episodes, the hemodynamic effects may have minimal to no impact on the left ventricle perfusion and, consequently, on transvalvular gradients.

Second, the choice of sedative agents used with MAC—particularly propofol versus dexmedetomidine—exhibited distinct cardiovascular effects that may influence transvalvular gradients differently. Propofol, commonly used in both MAC and GA, is known for its dose-dependent cardio-depressive effects, including its reduction in myocardial contractility and in systemic vascular resistance (SVR), which can exacerbate hypotension and potentially impact transvalvular gradients [30,31]. Studies comparing propofol and dexmedetomidine in cardiac surgery patients have consistently demonstrated propofol’s association with greater hemodynamic depression, including significant reductions in mean arterial pressure and cardiac output [32,33]. Conversely, dexmedetomidine, an alpha-2 adrenergic agonist, has been shown to provide more hemodynamic stability, reduce norepinephrine requirements, and better preserve myocardial contractility [34,35,36]. Despite these differences, both agents are widely used in TAVI sedation, and their effects on transvalvular gradients may vary depending on patient-specific factors and dosing strategies [37,38]. Given our institutional preference for dexmedetomidine-based MAC, the current study included only patients who received dexmedetomidine infusions. Despite dexmedetomidine’s favorable hemodynamic profile, the increase in transvalvular gradients at 24 h post-TAVI remained statistically comparable to the GA group, suggesting that anesthesia-related low-flow states may not be the primary factor influencing post-TAVI gradient changes.

Consistent with previous studies, the current investigation observed a statistically significant increase in 24 h transvalvular gradients in both the GA and MAC groups. These findings suggest that the observed changes are likely independent of the anesthesia technique or the specific pharmacodynamics of the sedative agents administered. The increase in gradients within the first 24 h post-TAVI may be attributed to several other factors. For example, patients with severe aortic stenosis have been shown to experience improvements in endothelial function following TAVI [39,40]. Changes in endothelial function post-procedure may contribute to the alterations in transvalvular gradients observed. Additionally, microvascular dysfunction, commonly found in patients with severe aortic stenosis [41,42], may impair coronary perfusion, myocardial oxygenation, and overall cardiac function, potentially influencing the gradient changes observed during the 24 h post-TAVI period. Although TAVR effectively reduces the severity of aortic stenosis, patient-specific variations in endothelial and microvascular responses could account for the differences in gradient dynamics. Further research is warranted to explore these factors in greater detail.

We acknowledge several limitations in this study. The retrospective design introduces the potential for selection bias and unmeasured confounding factors. Additionally, differences in baseline comorbidities between the GA and MAC groups, reflective of the evolving clinical practice and patient selection over time, may limit the direct comparability of these groups. As a single-center study, the findings may lack generalizability to other institutions with differing anesthesia and procedural practices. The exclusive use of dexmedetomidine for MAC at our institution also limits the applicability of the results to centers that routinely employ propofol-, remifentanil-, or ketamine-based MAC techniques for TAVI sedation. Furthermore, the study focused on short-term (24 h) transvalvular gradient changes, and longer-term follow-up may be necessary to fully assess the clinical significance of the gradient changes observed. While transvalvular gradients were assessed following ASE guidelines, interobserver variability remained a potential limitation. Lastly, patients requiring crossover from MAC to GA were excluded, potentially introducing bias, as this subgroup may represent higher-risk patients with unique hemodynamic challenges.

## 5. Conclusions

Despite the theoretical advantages of MAC in reducing anesthesia-induced low-flow states, this study found no significant difference in the magnitude of post-procedural transvalvular gradient increases between GA and MAC groups. The findings suggest that other physiological mechanisms, including endothelial function changes and microvascular dysfunction, may contribute to early post-TAVI gradient increases. Future studies should explore these alternative factors and assess their long-term impact on transvalvular gradients and clinical outcomes.

## Figures and Tables

**Table 1:** Baseline clinical characteristics of the patients.

	General Anesthesia (*n* = 201)	Monitor Anesthesia Care (*n* = 543)	*p*-Value
Age (years)	78 [71–84]	77 [71–83]	0.335
Gender (male)	118 (58.7%)	303 (55.8%)	0.48
Clinical history			
Diabetes mellitus	105 (52.2%)	225 (41.3%)	0.008
Hypertension	188 (93.5%)	477 (87.5%)	0.02
Hyperlipidemia	176 (87.6%)	460 (84.4%)	0.28
Peripheral vascular disease	93 (46.3%)	99 (18.2%)	<0.001
Stroke/TIA	39 (19.4%)	62 (11.4%)	0.004
COPD	57 (28.4%)	86 (15.8%)	<0.001
Atrial fibrillation	73 (36.3%)	151 (27.7%)	0.02
Previous permanent pacemaker	24 (11.9%)	63 (11.6%)	0.89
CKD (any stage)	121 (60.2%)	208 (38.2%)	<0.001
CKD stage 4 or ESRD	28 (13.9%)	39 (7.2%)	0.004
GFR	54 [40–70]	68 [50–85]	<0.001
Coronary artery disease	153 (76.1%)	301 (55.2%)	<0.001
Previous myocardial infarction	67 (33.3%)	59 (10.8%)	<0.001
Previous CABG	57 (28.4%)	71 (13%)	<0.001
Obstructive sleep apnea	44 (21.9%)	109 (20%)	0.57
BMI (kg/m^2^)	29 [25–34.94]	29 [25.1–34.4]	0.987
NYHA class III-IV symptoms	184 (92%)	442 (81.1%)	0.004
CHA2DS2-VASc score	5 [4–6]	4 [3–5]	<0.001
Post-TAVI length of stay (days)	3 [2–5]	1 [1–2]	<0.001

Abbreviations: TIA, transient ischemic attack; COPD, chronic obstructive pulmonary disease; CABG, coronary artery bypass graft; BMI, body mass index; NYHA, New York Heart Association; TAVI, transcatheter aortic valve implantation; ESRD, end-stage renal disease; and GFR, glomerular filtration rate. Data are expressed as the median [IQR, 25th–75th percentile] or proportion (percentages). *p* < 0.05 indicates that the difference between the two groups is statistically significant.

**Table 2:** Hemodynamics and anesthesia.

	General Anesthesia (*n* = 201)	Monitor Anesthesia Care (*n* = 543)	*p*-Value
Volume status			
Pre-TAVI weight (kg)	85.3 [71.1–99.8]	83.9 [71.2–99.3]	0.697
Post-TAVI weight (kg)	86.2 [71.6–100.7]	83.2 [70.9–98.4]	0.312
Pre-TAVI systolic blood pressure (mmHg)	149 [129–166]	154 [133–174]	0.1
Pre-TAVI diastolic blood pressure (mmHg)	65 [57–74]	71 [63–82]	<0.001
Immediate post-TAVI systolic blood pressure (mmHg)	128 [114–144]	124 [112–139]	0.017
Immediate post-TAVI diastolic blood pressure (mmHg)	53 [46–64]	59 [51–67]	<0.001
The 24 h post-TAVI systolic blood pressure (mmHg)	126 [117–139]	131 [118–142]	<0.001
The 24 h post-TAVI diastolic blood pressure (mmHg)	57 [50–64]	63 [56–70]	<0.001
Peri-procedural fluid (mL)	700 [300–950]	500 [250–700]	<0.001
The 24 h post-TAVI fluid balance (mL)	742.1 [98.2–1624]	494 [−64.3–1013]	<0.001
Medications			
Required vasopressors peri-procedurally	159 (79.1%)	194 (35.6%)	<0.001
Total fentanyl dose (mg)	250 [150–500]	0 [0–75]	<0.001
Total midazolam dose (mg)	2 [0–3]	0 [0–1]	<0.001
Propofol bolus dose (mg)	0 [0–50]	0 [0–0]	<0.001
Propofol infusion total (mg)	0 [0–77.6]	0 [0–0]	<0.001
Total propofol received during TAVI (mg)	50 [0–104]	0 [0–0]	<0.001
Dexmedetomidine bolus dose (mg)	0 [0–0]	70 [33.5–99.7]	<0.001
Dexmedetomidine infusion total (mg)	0 [0–0]	51.9 [30.6–83.3]	<0.001
Total dexmedetomidine received during TAVI (mg)	0 [0–0]	116.6 [79.9–160]	<0.001

Abbreviations: TAVI, transcatheter aortic valve implantation. Data are expressed as the median [IQR, 25th–75th percentile] or proportion (percentages). *p* < 0.05 indicates that the difference between the two groups is statistically significant.

**Table 3:** Echocardiographic characteristics of patients.

	General Anesthesia (*n* = 201)	Monitor Anesthesia Care (*n* = 543)	*p*-Value
Pre-TAVI			
Left ventricular ejection fraction (%)	55 [50–60]	60 [55–65]	<0.001
Aortic valve area (cm^2^)	0.8 [0.68–0.90]	0.79 [0.67–0.90]	0.495
Mean aortic valve gradient (mmHg)	41 [30.1–48]	39.2 [29.8–45]	0.073
Aortic regurgitation (moderate to severe)	10 (5%)	12 (2.2%)	0.13
Immediate Post-TAVI			
Mean transvalvular gradient (mmHg)	4.2 [3–6]	4.4 [3.2–6.1]	0.21
Changes 24 h Post-TAVI			
Mean transvalvular gradient (mmHg)	10 [7.4–13.7]	10.4 [7–13]	0.192
Effective orifice area (cm^2^)	1.7 [1.5–2.0]	1.62 [1.39–2]	0.02
Left ventricular ejection fraction (%)	56 [53.4–60]	61.4 [56–66]	<0.001
Presence of paravalvular leak (trace to mild)	37 (18.4%)	26 (4.8%)	<0.001
Presence of paravalvular leak (moderate to severe)	1 (0.5%)	1 (0.2%)	0.47
Gradient Changes in 24 h			
Mean transvalvular gradient after 24 hr increase (mmHg)	5.8		<0.001
Mean transvalvular gradient after 24 hr increase (mmHg)		6	<0.001
Meant transvalvular gradient delta (mmHg)	5.1 [3–8.1]	5.8 [3.2–9]	0.139

Abbreviations: TAVI, transcatheter aortic valve implantation. Data are expressed as the median [IQR, 25th–75th percentile] or proportion (percentages). *p* < 0.05 indicates that the difference between the two groups is statistically significant.

## Data Availability

The data presented in this study are available upon request from the corresponding author.

## Abbreviations

The following abbreviations are used in this manuscript:

AKI	Acute kidney injury
ASE	American Society of Echocardiography
BIS	Bispectral index
f	Frequency (f)
GA	General anesthesia
IQR	Interquartile ranges
LVEF	Left ventricular ejection fraction
LVOT	Left ventricular outflow tract
MDN	Medians
NPO	Nil per os
SAVR	Surgical aortic valve replacement
SVR	Systemic vascular resistance
TAVI	Transcatheter aortic valve implantation
TTE	Transthoracic echocardiogram (TTE)
